# Supplementary figures and images for: Predicting bioprocess targets of chemical compounds through integration of chemical-genetic and genetic interactions
Source: PLoS Comput Biol. 2018 Oct 30;14(10):e1006532. doi: 10.1371/journal.pcbi.1006532 (PMC6226211; doi:10.1371/journal.pcbi.1006532)

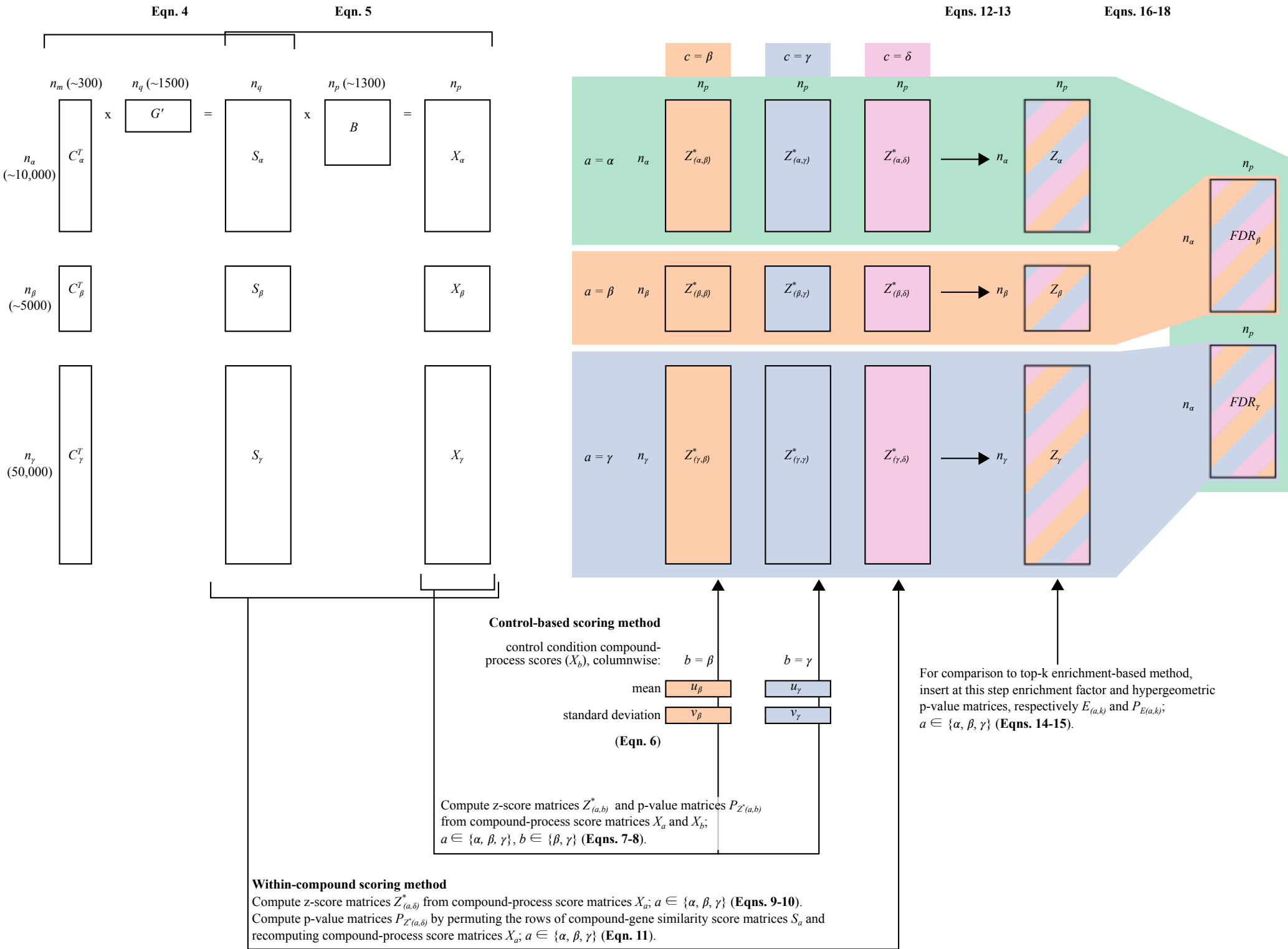

Supplement: S1 Fig — Further details on the presented procedures, including equations, are given in “Predicting the biological processes perturbed by compounds” in Materials and Methods. (PDF) [file pcbi.1006532.s001.pdf]

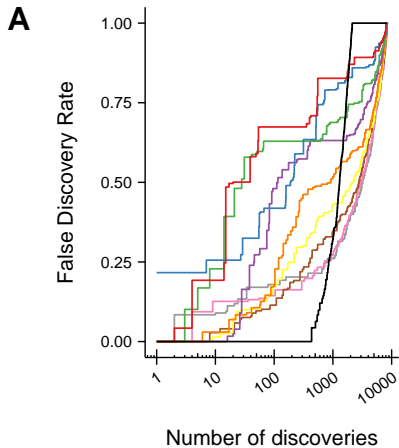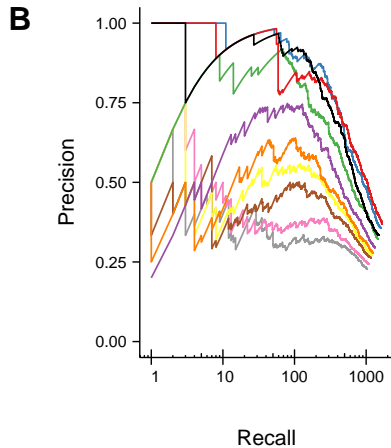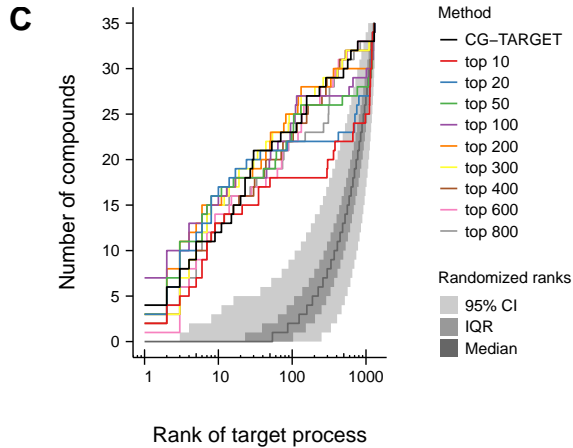

Supplement: S2 Fig — Perturbed biological processes were predicted using both CG-TARGET and methods that calculated enrichment on the set of each compound’s n most similar genetic interaction profiles (“top n,” n ∈ {10, 20, 50, 100, 200, 300, 400, 600, 800}). (A) Bioprocess prediction false discovery rate estimates derived from resampled chemical-genetic interaction profiles, performed on compounds from the RIKEN dataset. (B) Precision-recall analysis of the ability to recapitulate gold-standard annotations within the set of top bioprocess predictions for ~4500 simulated compounds. Each simulated compound was designed to target one query gene in the genetic interaction network and thus inherited gold-standard bioprocess annotations from its target gene. (C) For each of 35 well-characterized compounds in the RIKEN dataset with literature-derived, gold-standard bioprocess annotations, we determined the rank of its gold-standard bioprocess within its list of predictions. The number of compounds for which a given rank (or better) was achieved is plotted. The grey ribbons represent the median, interquartile range (25th to 75th percentiles), and 95% confidence interval of 10,000 rank permutations. (PDF) [file pcbi.1006532.s002.pdf]
