# Supplementary material for: Predicting bioprocess targets of chemical compounds through integration of chemical-genetic and genetic interactions
Source: PLoS Comput Biol. 2018 Oct 30;14(10):e1006532. doi: 10.1371/journal.pcbi.1006532 (PMC6226211; doi:10.1371/journal.pcbi.1006532)
Supplement: S3 Fig — Each term was evaluated using precision-recall statistics (area under the precision-recall curve divided by the area under a curve produced by a random classifier) to analyze its ability to rank simulated chemical-genetic interaction profiles from which it was annotated as a gold-standard bioprocess. Green nodes represent the 100 best-performing GO biological process terms, yellow nodes represent terms for which predictions were made but did not rank among the top 100, and white nodes represent terms in the Biological Process ontology that were not selected for bioprocess prediction. Hovering the mouse over each node reveals its GO ID and name. (HTML) [file pcbi.1006532.s003.html]

Top 100 GO terms by AUPR over background ratio
