# Supplementary material for: Predicting bioprocess targets of chemical compounds through integration of chemical-genetic and genetic interactions
Source: PLoS Comput Biol. 2018 Oct 30;14(10):e1006532. doi: 10.1371/journal.pcbi.1006532 (PMC6226211; doi:10.1371/journal.pcbi.1006532)
Supplement: S4 Fig — Same as S3 Fig, but for the 100-worst performing GO biological process terms. (HTML) [file pcbi.1006532.s004.html]

Bottom 100 GO terms by AUPR over background ratio
