# Supplementary material for: Predicting bioprocess targets of chemical compounds through integration of chemical-genetic and genetic interactions
Source: PLoS Comput Biol. 2018 Oct 30;14(10):e1006532. doi: 10.1371/journal.pcbi.1006532 (PMC6226211; doi:10.1371/journal.pcbi.1006532)
Supplement: S5 Table — Any instance of the symbols a, b, or c should be expanded into individual expressions for each of the members of those respective sets (i.e. Ca becomes Cα, Cβ, and Cγ). Where two of these symbols appear in a variable’s subscript, that variable exists for all pairwise combinations of those set members. (DOCX) [file pcbi.1006532.s009.docx]

Table S5. Reference for variables and symbols used to describe the CG-TARGET method in Materials and Methods.

Any instance of the symbols *a*, *b*, or *c* should be expanded into individual expressions for each of the members of those respective sets (i.e. *C_a_* becomes *C_α_*, *C_β_*, and *C_γ_*). Where two of these symbols appear in a variable’s subscript, that variable exists for all pairwise combinations of those set members.

| Profile and statistic types | | Description |
| --- | --- | --- |
| *α* | | Derived from treatment conditions |
| *β* | | Derived from negative control conditions |
| *γ* | | Derived from randomly resampled treatment profiles |
| *δ* | | Derived by shuffling each compound’s gene similarity scores (“within-profile” stats) |
|  | |  |
| Sets | | Description |
| *a* ∈{*α, β, γ*} | | All profiles |
| *b* ∈{*β, γ*} | | Control profiles |
| *c* ∈{*β, γ, δ*} | | All statistic types (2 “control-derived” and 1 “within-profile”) |
|  |  |  |
| Matrices | Dimensions | Description |
| *C­_a_* | *n_a_* x *n_m_* | Chemical-genetic interaction profiles |
| *G* | *n_m_* x *n_q_* | Genetic interaction profiles |
| *B* | *n_q_* x *n_p_* | Bioprocess annotations |
| *S_a_* | *n_a_* x *n_q_* | Compound-gene similarity scores |
| *X_a_* | *n_a_* x *n_p_* | Compound-process scores (sum of gene similarity scores to process members) |
| *Z^*^*_(_*_a_*_,_ *_c_*_)_ | *n_a_* x *n_p_* | Compound-process z-scores derived from individual statistic types |
| *P_Z*_*_(_*_a_*_,_ *_c_*_)_ | *n_a_* x *n_p_* | Compound-process p-values derived from individual statistic types |
| *Z_a_* | *n_a_* x *n_p_* | Final compound-process z-scores |
| *P_a_* | *n_a_* x *n_p_* | Final compound-process p-values |
|  |  |  |
| Vectors | Size |  |
| *u_b_* | *n_p_* | Mean of each process’ compound-process scores (controls only) |
| *v_b_* | *n_p_* | Standard deviation of each process’ compound-process scores (controls only) |
