# Supplementary material for: Predicting bioprocess targets of chemical compounds through integration of chemical-genetic and genetic interactions
Source: PLoS Comput Biol. 2018 Oct 30;14(10):e1006532. doi: 10.1371/journal.pcbi.1006532 (PMC6226211; doi:10.1371/journal.pcbi.1006532)
Supplement: S1 Data — All supporting datasets are available from the Dryad digital repository at the following link: https://dx.doi.org/10.5061/dryad.nr2cf12. They represent all raw data and final results for the application of CG-TARGET to the RIKEN chemical-genetic interaction screen. (DOCX) [file pcbi.1006532.s010.docx]

**S1 Data. Descriptions of supporting datasets uploaded to the Dryad digital repository.** All supporting datasets are available from the Dryad digital repository at the following link: https://dx.doi.org/10.5061/dryad.nr2cf12. They represent all raw data and final results for the application of CG-TARGET to the RIKEN chemical-genetic interaction screen.

**All chemical-genetic interaction profiles.** Chemical-genetic interaction profiles across 289 diagnostic *S. cerevisiae* deletion mutants for 8418 compounds, 5724 negative experimental controls, and 50,000 randomly resampled profiles from the RIKEN chemical-genetic interaction screen. Profiles consist of z-scores that represent the deviation of observed from expected mutant barcode abundance measured from pooled screens.

**Genetic interaction profiles.** Genetic interaction profiles for 1505 query gene mutants crossed into an array of 3841 mutants in *S. cerevisiae*. Profiles consist of epsilon scores that represent the deviation of the observed double mutant fitness from the expected fitness derived from the single mutant fitness values using a multiplicative model. Epsilon scores were set to zero if their magnitudes or significance values did not meet specific criteria (|*ϵ*| < 0.08 or *p* ≥ 0.05). These profiles are provided prior to the *L*_2_-normalization performed in the CG-TARGET method.

**Gene Ontology biological process terms.** Annotation of *S. cerevisiae* genes to Gene Ontology biological process terms. Both the *S. cerevisiae* annotations to biological process terms and the ontology itself were downloaded on September 12, 2013 using Bioconductor in R. Terms were propagated from child to parent terms using “is_a” relationships. The genes included in this matrix are the union of the genetic interaction profile “query” genes and the diagnostic mutant array, enabling the reproduction of analyses based on either similarity to genetic interaction profiles or on interaction scores directly. Terms are not filtered by size.

**All compound-bioprocess predictions.** Complete set of perturbed bioprocess predictions, computed using CG-TARGET, for 8418 compounds, 5724 negative experimental controls, and 50,000 randomly resampled profiles from the RIKEN chemical-genetic interaction screen.

**Bioprocess predictions for compounds, FDR ≤ 25%.** Same as “All compound-bioprocess predictions” dataset but filtered to include results only for compounds and with false discovery rate ≤ 25%. This dataset is provided for convenience since many of our analyses were performed just on the set of high-confidence predictions.
